# Effect of Evening Primrose (*Oenothera biennis*) Oil Cake on the Properties of Polyurethane/Polyisocyanurate Bio-Composites

**DOI:** 10.3390/ijms22168950

**Published:** 2021-08-19

**Authors:** Joanna Paciorek-Sadowska, Marcin Borowicz, Marek Isbrandt

**Affiliations:** Department of Chemistry and Technology of Polyurethanes, Institute of Materials Engineering, Kazimierz Wielki University, 30 Chodkiewicza Street, 85-064 Bydgoszcz, Poland; m.isbrandt@ukw.edu.pl

**Keywords:** *Oenothera biennis*, evening primrose, oil cake, bio-filler, polyurethane/polyisocyanurate foam, foam properties

## Abstract

Rigid polyurethane/polyisocyanurate (RPU/PIR) foam formulations were modified by evening primrose (*Oenothera biennis*) oil cake as a bio-filler in the amount of 5 to 50 wt.%. The obtained foams were tested in terms of processing parameters, cellular structure (SEM analysis), physico-mechanical properties (apparent density, compressive strength, brittleness, accelerated aging tests), thermal insulation properties (thermal conductivity coefficient, closed cells content, absorbability and water absorption), flammability, smoke emission, and thermal properties. The obtained results showed that the amount of bio-filler had a significant influence on the morphology of the modified foams. Thorough mixing of the polyurethane premix allowed better homogenization of the bio-filler in the polyurethane matrix, resulting in a regular cellular structure. This resulted in an improvement in the physico-mechanical and thermal insulation properties as well as a reduction in the flammability of the obtained materials. This research provided important information on the management of the waste product from the edible oil industry and the production process of fire-safe RPU/PIR foams with improved performance properties. Due to these beneficial effects, it was found that the use of evening primrose oil cake as a bio-filler for RPU/PIR foams opens a new way of waste management to obtain new “green” materials.

## 1. Introduction

Evening primrose is a common plant belonging to the *Onagraceae* family [1]. It is mainly found in the temperate climate zone of North America, but can also be found in tropical climates. This plant is in the form of annual, biennial, or perennial herbs with alternating and mostly narrow leaves. *Oenothera* species are known for their white, pink, yellow, and red saucer-shaped and mostly fragrant flowers [2]. Several of its species are currently cultivated in Europe, North America, and New Zealand. The main purpose of cultivating this plant is primarily to obtain oil from its seeds [3,4].

The world production of oilseeds and their processing products shows an upward trend with slight fluctuations depending mainly on weather conditions. Over the last 10 years, the total value of vegetable oil production has increased by nearly 80%, reaching 185.7 million tons in the 2016/2017 season, over 4% higher than in the previous year. Moreover, production is still forecast to increase to 200.0 million tons in 2023 [5].

The by-product in the production of vegetable oils is degreased pomace, the so-called cake. These are classified as organic residues of various nutritional, energy, and fertilizing value, often characterized by unfavorable pH. Due to their composition, they constitute an ecological hazard during unskillful storage and a significant risk factor for the occurrence of putrefactive processes. Therefore, they become a difficult economic and environmental problem. So far, the proposed solutions to this ecological and economic problem are incomplete and do not solve it comprehensively [6]. In 2014/2015, the global oil industry produced 279 million tons of meal and cake, at the same time noting a 57% increase in relation to the years 2000–2004. Forecasts assume that by 2024, the total production volume of these by-products will reach 355 million tons [7].

With slight changes in the consumption of evening primrose oil in households, there will be a further increase in the generation of oil cake in the food and pharmaceutical industries. One of the solutions is the use of oil cakes for energy purposes. However, their use for energy purposes is not a good solution. Oil cakes are characterized by a large variety of thermally easily degradable organic compounds (e.g., proteins, sulfur compounds). Therefore, the correct combustion of these substances must take into account the possibility of the formation of tar products during the pyrolysis process as well as the formation of soot [8].

Early development of innovative methods of cake utilization will prevent possible problems with their overproduction. Hence the concept of our work, consisting in the use of evening primrose cake as a bio-filler in the production of polyurethane composites.

Polyurethanes (PUs) are the most interesting polymers in terms of their variety of applications. These materials can be used in the form of rigid, semi-rigid, and flexible foams, elastomers, coatings, and adhesives [9,10,11]. One of the most important groups of polyurethane materials are rigid polyurethane foams. Their low density and thermal conductivity combined with their mechanical properties make them excellent thermal and acoustic insulators and construction materials. PU production is still largely dependent on crude oil despite its wide range of applications. In the face of new, pro-environmental trends, the PU industry has adapted to the increasingly stringent legal regulations and stringent consumer requirements over the past few years. Light, resistant, and versatile polyurethane materials contribute to saving energy and natural resources in strategic sectors of the economy such as civil engineering, health care, automotive, and transport. Following new trends in sustainable development, more and more research is focused on replacing polyols (the main petrochemical components in PU systems) by renewable hydroxyl derivatives obtained from natural oils [12,13,14,15,16,17,18]. 

Another sustainable approach in the synthesis of polyurethane materials is their modification with fillers from recycled renewable sources. The use of such fillers (bio-fillers) is one of the ways of fitting into the doctrines of green chemistry and the circular economy in the synthesis of polyurethanes. Research on the use of waste bio-fillers in the production of PUs is carried out by many researchers around the world. This is due to the necessity to develop methods for the proper preparation of bio-fillers for the reaction, develop appropriate formulations, evaluate the impact of the raw materials used on processing, the properties of composites, and to find an application for the obtained materials. The polyurethane materials were modified by bio-fillers such as potato starch [19], cellulose [20,21], curcumin [22], keratin feathers [23], shells walnut [24], and date palm particles [25]. The addition of low-cost plant fillers into the polymer matrix allows for the improvement of selected properties of the obtained foam materials, a reduction in their price as well as the production of environmentally friendly composites.

The aim of the research described in this paper was to determine the effect of evening primrose oil cake (EPC) on the properties of rigid polyurethane/polyisocyanurate (RPU/PIR) foams. Evening primrose cake, after appropriate preparation, could be used as a physical bio-filler in the formulation of RPU/PIR foams. The article also presented a composition optimization of this filler in a polyurethane formulation in order to assess the possibility of modifying the structure and functional properties of RPU/PIR foams.

## 2. Results and Discussion

### 2.1. Foaming Process

In designing the optimal composition of RPU/PIR foams, it is helpful to analyze the foaming process based on the results of processing times (cream, string gel, tack free, and free rise times). Analysis of the foaming parameters allowed us to determine the reactivity of the polyol premix. Detailed knowledge of the process parameters during the synthesis of polyurethane foams is very important when modifying the formulation by physical fillers. Several-fold increase in the volume of the reaction mixture was observed during the foaming process and affected the formation of the cellular structure. In turn, the size and shape of the cells determine the mechanical and thermal insulation properties of the obtained foams [26]. The results of processing times measured during the obtaining of RPU/PIR are presented in Table 1.

The use of EPC for the production of RPU/PIR foams resulted in changes in the values of the processing times of the modified foams in comparison to the reference foam. A slight elongation in the string gel (from 32 s for EPC0 to 38 s for EPC50 foam), tack free (from 74 s for EPC0 to 98 s for EPC50 foam), and free rise (from 66 s for EPC0 to 83 s for EPC50 foam) times was noted after using the evening primrose oil cake. However, the cream time did not change and was 16 s for all foams. In the case of the obtained RPU/PIR foams modified by EPC, the positive effect was a relatively small elongation in all processing times, even at 50 wt.% filling. This proved the correct selection of the quantitative and qualitative composition of the raw materials in the RPU/PIR foam formulation and sufficient mixing of the polyol premixes.

Usually, the use of fillers contributes to a significant increase in these parameters. Similar observations were made by Gu et al. [27], Prociak et al. [28] and Kuźnia et al. [29]. The obtained values of processing times during the use of EPC for the synthesis of RPU/PIR foams were close to the results obtained during research on the use of rapeseed cake in a polyurethane formulation [30]. The observed slight elongation in processing times occurred despite the use of the same amount of distilled water in all the formulations. This was due to the fact that small solid filler particles were evenly dispersed in the polyol premixes during the nucleation process and contributed to more intensive bubble formation. The formation of new bubbles led to an elongation in the string gel, tack free, and free rise times. A similar effect of fillers on the foaming process was observed by Cao et al. [31], Fan et al. [32], and Onuegbu et al. [33]. On the other hand, the growth of newly formed bubbles may be hampered by the increased viscosity of the system modified with bio-filler. This is also important for the obtained values of reaction times. The increasing content of EPC in polyurethane systems contributed to the reduction in the mobility of molecules and the elongation in the reaction time between the polyol raw material and isocyanate raw material. The used bio-filler slowed down the curing of the polyurethane structure, extending the gelation (tack free) time of the obtained materials.

### 2.2. Cell Structure and Thermal Insulation Properties of RPU/PIR Foams

**Figure 1:**
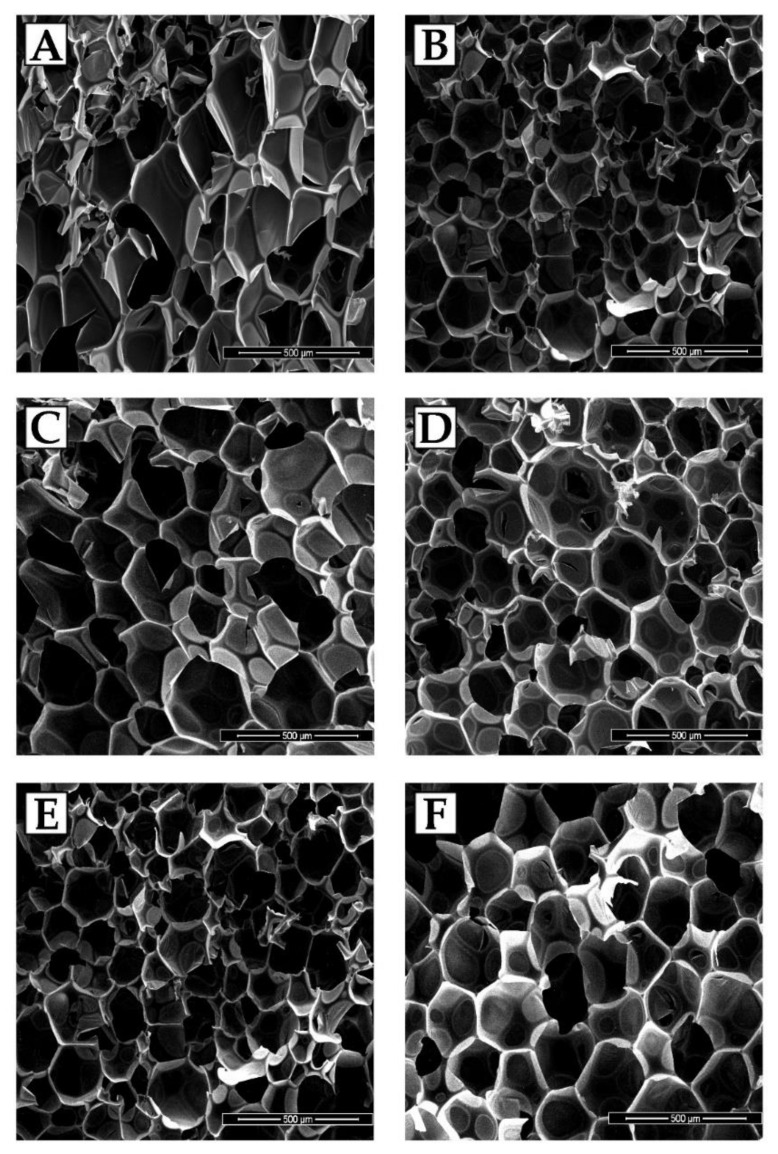
Cell structures of selected RPU/PIR foams: (**A**) EPC0, (**B**) EPC10, (**C**) EPC20, (**D**) EPC30, (**E**) EPC40, (**F**) EPC50.

The cellular structure of the RPU/PIR foams depends on many factors. The following parameters play a fundamental role in shaping this structure: premix viscosity, their modification by fillers, and method of foaming. In typical polyurethane foams with an apparent density of 30–40 kg/m^3^, the blowing agent constitutes about 95% of the material volume. Appropriate selection of the type of blowing agent in the foam formulation (physical or chemical) and the foaming method is aimed at obtaining a regular structure of the polyurethane matrix and the best possible thermal insulation properties. The chemical foaming method was used in the conducted research. The synthesized RPU/PIR foams were foamed by carbon dioxide generated in the reaction of isocyanate groups with distilled water. Micrographs of the cell structure of selected RPU/PIR foams are shown in Figure 1.

RPU/PIR foams with EPC were characterized by a spherical shape of the cells in comparison with the reference foam without the bio-filler. The EPC0 foam cells had a more elongated (anisotropic) shape. In the case of foams modified by the bio-filler, the more spherical (isotropic) shape of the cells could have been influenced by the nucleation by filler particles and the elongated gelation of the foam. However, elongation in the gelation of foam is not an entirely beneficial effect, because it may disturb the balance between the gelling and crosslinking reactions during foaming of the PU composition. As a result of this, cells with an elongated shape in the direction of the foam rise can be obtained. This structure adversely affects the mechanical properties of the foams [34]. It can be seen in Figure 1A–F that the obtained foams had a closed cell structure. This is typical for rigid polyurethane foams and is desirable for most applications of these materials. The presence of closed-cell walls results in low water absorption and low moisture permeability as well as enclosing the blowing agent inside the foam. This directly affects the low thermal conductivity of the obtained material. The EPC0 foam was characterized by cells elongated in the direction of rise. It can be seen in Figure 1A that the cells of EPC0 foam were thin-walled and vertically oriented. The reason for this was the lower viscosity of the reference polyol premix and easier expansion of the cells in the direction of foam rise. Some cell walls of the EPC0 foam showed a tendency to crack and break. On the other hand, the cell structure of the foams modified by EPC (Figure 1B–F) was more regular with spherical cells. Increasing the content of bio-filler resulted in obtaining structures with smaller cells that were evenly distributed. In the case of a few cells, broken walls were observed (mostly due to mechanical damage during sample preparation). Bio-filler particles built into the walls of the cells, strengthening them, and thus making them more resistant to mechanical damage.

Increasing content of EPC in the polyurethane formulation improved the interfacial adhesion between the bio-filler particles and the polyurethane matrix. It led to a more effective formation of the polyurethane structure. The higher cross-linking degree of RPU/PIR foams modfied by EPC could prevent cell disruption during the foaming process. Moreover, the bio-filler particles could act as nucleation places for cell formation in the stage of forming the polyurethane structure. Increasing EPC content caused more cells to start nucleating at the same time. Therefore, the foam had a higher number of spherical cells with decreased diameter and thicker walls. The addition of a bio-filler could promote the formation of a large number of small cells. The same dependence was also observed by Faruk et al. [35] and Luo et al. [36]. The foams modified by a bio-filler had a smaller cell diameter and thicker cell walls in comparison with the EPC0 reference foam. The cell diameter decreased from 396 µm for the EPC0 foam to 220 µm for the EPC50 foam (with the highest content of EPC). This effect was related to the introduction of a bio-filler into the polyurethane cell walls, which increased their thickness and at the same time made them stiff and stronger. The results of the statistical analysis of the SEM micrographs of the EPC0, EPC10, EPC20, EPC30, EPC40, and EPC50 foams (i.e., average cell diameter, average wall thickness, and average content of cells per area unit) are presented in Table 2.

It can be concluded that modification of rigid RPU/PIR foams by evening primrose oil cake increased the content of closed cells in the obtained bio-composites. All modified RPU/PIR foams were characterized by a high value of this parameter (approx. 90%). The high content of closed cells and the uniform polyurethane structure made it possible to obtain foams with good thermal insulation properties. The parameters used to evaluate this property are: the thermal conductivity coefficient (λ), the content of closed cells, absorbability (A), and water absorption (WA) of the polyurethane matrix. The results of the tests of these parameters are presented in Table 3.

**Figure 2:**
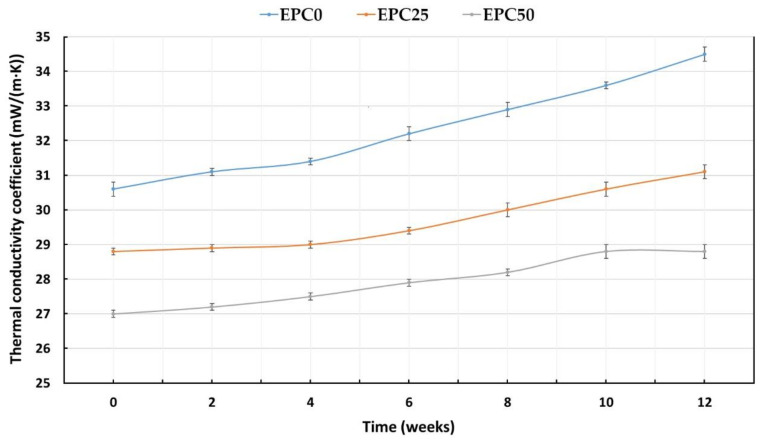
Changes in the value of the thermal conductivity coefficient of selected foams during the aging process at room temperature.

The thermal conductivity coefficient (λ) is one of the most important parameters that determines the possible use of polyurethane foams as a thermal insulation material. Thermal conductivity is influenced by several parameters such as closed cell content, cell size, and absorbability and water absorption [37,38]. It can be concluded on the basis of the test results presented in Table 3 that the value of the thermal conductivity coefficient decreased from 34.6 mW/(m·K) for the EPC0 reference foam to 27.4 mW/(m·K) for the foam with the highest amount of EPC. The λ value decreased with the increase in the content of bio-filler in the polyurethane matrix. This means that the thermal insulation properties of the modified RPU/PIR foams were improved. The addition of the bio-filler led to the formation of a more uniform structure with smaller cells, as shown in Figure 1A–F. Such a structure significantly reduced the transfer of thermal radiation through the cell walls, and thus the thermal conductivity of the polyurethane foams. Typically, the addition of bio-fillers to the polyurethane formulation increases the thermal conductivity of PU foams. This is due to the formation of an inhomogeneous cell structure and higher content of open cells. This was observed by Wu et al. [39] during their research. The authors showed that the thermal conductivity of the modified foams increased during the addition of chicken feather as a bio-filler. The addition of feathers resulted in a significant reduction in the content of closed cells and damage to its structure, which at the same time made it easier to transfer heat. However, when using EPC as a filler for RPU/PIR foams, an increase in the content of closed cells from 80.9% for EPC0 to over 93.1% for the foam with the highest content of bio-filler was observed. A study of the change in the thermal conductivity coefficient of selected foams over 12 weeks was carried out to assess the thermal conductivity behavior in the long-term. The obtained results are shown in Figure 2.

The most stable value of the thermal conductivity coefficient was shown by the foam containing the highest amount of bio-filler (EPC50). The behavior of the thermal conductivity coefficient depends on the rate of diffusion of the blowing agent from the foam material. It is mainly related to the cross-linking density of the polyurethane matrix and the content of closed cells. The distribution of the EPC in the matrix of the obtained foams contributed to the creation of a stronger and more precisely packed polymer structure. This minimized the outflow of carbon dioxide to the outside (λ_CO2_ = 14.6 mW/(m⋅K)) and the inflow of air to the inside (λ_air_ = 24 mW/(m⋅K)). Thus, it increased the long-term preservation of the thermal conductivity coefficient. Galakhova et al. [40] reported that carbon dioxide leaves the rigid polyurethane foams (RPUfs) after 2.5 months. Carefully selected raw materials in the polyurethane formulation made it possible to develop spherical cells with undamaged walls. This was confirmed by the SEM analysis. Such characteristics of the cells contributed to the decrease in absorbability and water absorption from 10.9% for the EPC0 foam to 6.6% for the EPC50 foam and from 5.5% for the EPC0 foam to 1.8% for the EPC50 foam, respectively. Therefore, it can be concluded that the cellular morphology of the RPU/PIR foams was the dominant factor influencing the water sorption of the analyzed materials. The presence of the bio-filler in the material was another reason for the lower absorbability and water absorption of the obtained foams. EPC built into the polyurethane matrix made it possible to obtain a more ordered, stable structure that was more resistant to mechanical damage and cell disruption.

### 2.3. Physico-Mechanical and Aging Properties of RPU/PIR Foams

The apparent density of porous materials is one of the most important parameters, the value of which has a large influence on the physico-mechanical properties of the obtained materials (e.g., compressive strength or brittleness). The results of apparent density, compressive strength, brittleness, and simulated aging of the obtained foams are presented in Table 4.

**Figure 3:**
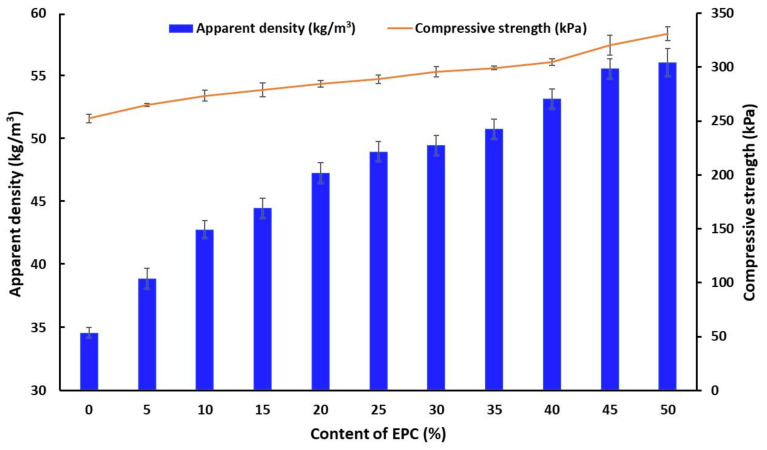
Dependence between the apparent density, compressive strength, and amount of EPC.

Insulating structures made from rigid polyurethane foams are exposed to compression and crushing, depending on the characteristics of the application. Therefore, a careful evaluation of these parameters in relation to the apparent density is very important. Typical RPUfs used in engineering construction have a density of approx. 30 kg/m^3^ [41]. The dependence between the apparent density and compressive strength RPU/PIR foams containing different amounts of EPC is shown in Figure 3.

It was found on the basis of the obtained test results that the compressive strength of the foams increased with the increase in the EPC content. The reference foam (EPC0) had the lowest density of 34.6 kg/m^3^ and the lowest compressive strength of 252.5 kPa, while the sample with the highest content of EPC had the maximum density of 56.1 kg/m^3^ and the highest compressive strength of 331.1 kPa. The main reason for such a trend was the composition of the polyurethane formulation. During this research, RPU/PIR foams were obtained using a chemical foaming method in which carbon dioxide was generated as a blowing agent. The composition of the solid phase was increased when adding EPC to the obtained foams while the same volume of carbon dioxide was generated. This resulted in an increase in the density of the obtained materials. Moreover, the mechanical properties of the foam are mainly related to its apparent density. Thus, the compressive strength increased with increasing apparent density of RPU/PIR foams.

It can be concluded based on the analysis of the test results that the mechanical properties also depended on the cell structure of polyurethane foams modified with EPC. As shown in Figure 1, the bio-filler addition contributed to the synthesis of RPU/PIR foams with a more homogeneous structure and a higher content of closed cells. This resulted in an increase in the compressive strength of the obtained materials. The reinforcing effect can also be attributed to the interfacial adhesion between the EPC and polyurethane matrix. This made it easier to transfer the stresses. In the case of the reference foam (EPC0), the structure was less homogeneous and the mechanical properties deteriorated. Moreover, it should also be noted that the ground EPC did not show a tendency to agglomerate. Agglomeration of particles in the polyurethane matrix leads to interfacial separation of the foam structure and promotes damage to the cellular structure under compressive load. A similar dependence was noted by Ciecierska et al. [42]. The authors of the article found that the addition of carbon nanotubes, as a filler, improved the mechanical strength by about 20%. This increase was attributed to the strengthening effect of the filler and a change in the foam structure (e.g., smaller cell sizes, greater homogeneity of structure, and uniform dispersion of the bio-filler).

The addition of EPC to RPU/PIR foams also contributed to the beneficial decrease in the brittleness of these materials. The brittleness of the reference foam was 23.8%. A decrease in this parameter to 13.1% was observed for the foam containing the highest bio-filler addition. The lower brittleness of the obtained polyurethane materials was related to the reduction in cell size, strengthening their walls by a filler and obtaining a compact structure, which was more resistant to mechanical damage.

There is also a correlation between the compressive strength and the stability of linear dimensions. The internal gas pressure in cells changes with temperature changes. It creates a differential pressure between the pressure in the foam cells and the external atmospheric pressure. This differential pressure must be less than the compressive strength of the foam to maintain dimensional stability. Deformation of the foam should not occur when the compressive strength is higher than 100 kPa. This value is higher than the possible differential pressure between the atmospheric pressure and the pressure inside the foam cells. It was found by comparing the results of aging tests for the obtained RPU/PIR foams that there was a correlation between the stability of linear dimensions, mass loss, changes of geometrical volume, and the use of EPC in the foam formulation. It was observed during the simulated aging of the samples that mass loss and the changes in geometrical volume did not exceed 3% for all modified foams. Similarly, changes in linear dimensions did not exceed 2.2%. Changes in the length, width, and mass of the foams after exposure to high temperature indicated smaller changes in these parameters after the application of bio-filler than for the reference foam. This meant that the bio-filler had a stabilizing effect on the obtained foams at high temperatures. This may suggest that EPC may be a good thermal stabilizer for RPU/PIR foams. The dimensional stability of modified foams (EPC5–EPC50 foams) improved by an average of 20% in comparison with the EPC0 foam. The observed changes in dimensional stability for each modified RPU/PIR foam were considered to be minor and within acceptable limits for an industrial application [43,44].

### 2.4. Flammability and Thermal Resistance of RPU/PIR Foams

Rigid polyurethane foams have excellent mechanical and thermal insulation properties, but also have some disadvantages. These include the high flammability and toxicity of gaseous products released during thermal decomposition and combustion [45]. The results of flammability and smoke emission tests of selected RPU/PIR foams carried out with the cone calorimeter and limiting oxygen index (LOI) method are presented in Table 5.

**Figure 4:**
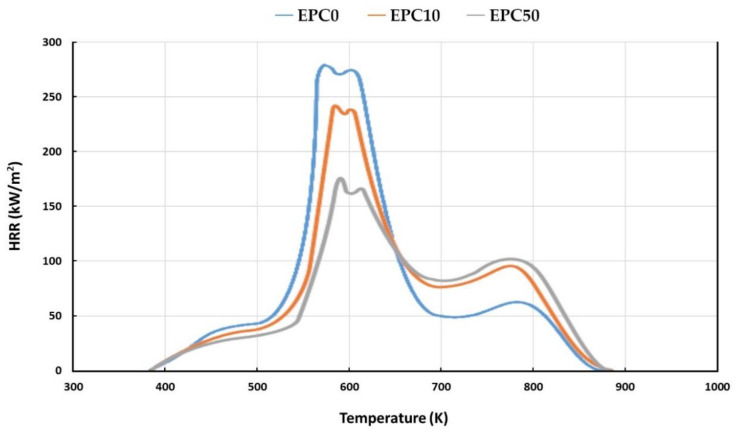
Dependence of the heat release rate (HRR) of selected RPU/PIR foams on temperature.

One of the important parameters considered in this research was the method of reducing the flammability of polyurethane materials while maintaining a low level of smoke emission. When analyzing the obtained test results on a cone calorimeter, it can be noticed that all times to ignition (TTI) were in range of 1–3 s, which was a result of the porous and organic structure of the tested materials. One of the key parameters used to assess the flammability of materials is the heat release rate (HRR). It has been proven that doubling the HRR of a material can lead to a more than threefold reduction in the survival time of fire victims [46]. A reduction in the value of maximum HRR for most of the obtained RPU/PIR bio-composites was noted as a result of this test. Comparing the results obtained for the reference foam with the values determined for the modified foams, it was found that the addition of a small amount of EPC resulted in a reduction in heat released during combustion. These observations may indicate the occurrence of a synergistic effect between the EPC and used flame retardant (TCMP). The lowest HRR values were obtained for the samples with the highest content of evening primrose oil cake (Figure 4).

The HRR curves of the selected RPU/PIR foams showed two decomposition peaks. The first occurred in the temperature range of 573–673 K, while the second at 773 K (Figure 4). The first peak was caused by the formation of a char layer on the surface of the foam during burning. The polyurethane matrix is exposed to a flame by pyrolysis of the char layer during the combustion process. This resulted in a second peak in HRR. The use of EPC shifted both peaks to higher temperatures. This indicated that the modified foams were more resistant to fire. This effect can be explained by the higher content of phosphorus and nitrogen in the polyurethane composition, introduced with the bio-filler into the PU formulation. This resulted in the formation of a char layer on the surface of the burning foam. Similar dependence regarding the effects of phosphorus and nitrogen on the reducing flammability of RPU/PIR foams was observed by Liu et al. [47]. Moreover, the decrease in HRR observed during the tests could also be the effect of a synergistic interaction between other additives. It was also found that the modified foams were characterized by a significantly lower mass loss (about two times lower for the foam with the highest EPC content) than the EPC0 foam. The reason for the decrease in flammability of foams containing bio-filler was the formation of a permanent char layer on the surface of modified foams, limiting heat and mass transfer between the material and the flame. A significant elongation in the time to reaching HRR_max_ (tHRR_max_) was observed. It was also caused by the formation of a char layer on the surface of the burning material. It can also be seen that the flammability of the modified RPU/PIR foams decreased with the increase in their apparent density. However, this was probably the effect of heat conduction through the sample and is irrelevant to determining the actual rate of pyrolysis. However, this indicated a significant change in the combustion behavior of the foam with increasing apparent density. Undoubtedly, the presence of both urethane bonds and isocyanurate rings in the foam composition had the effect of reducing the flammability of RPU/PIR foams. Isocyanurate rings are more thermally stable than urethane bonds from the thermodynamic point of view as urethane bonds dissociate at about 200 °C, while the isocyanurate rings at about 350 °C [20]. 

Smoke is a very serious fire hazard because most fire victims are poisoned by toxic gases. The cone calorimeter provides some information about the chemical composition of the smoke. The obtained information is the amounts of carbon monoxide and carbon dioxide that are generated during the combustion of the sample. The amounts of carbon monoxide and dioxide released during combustion of selected samples of the obtained materials are presented in Table 5. It can be seen that the addition of EPC significantly reduced the smoke emission. Smoke release may also be limited by a solid, phosphorus-rich and nitrogen-rich char layer that formed in the initial combustion phase. An important factor in assessing the possibility of limiting the fire risk is also the weight ratio of CO to CO_2_ in RPU/PIR foams [48] and determines the level of reaction of complete combustion. The higher CO/CO_2_ weight ratio, the lower the total combustion. Thus, the smoke emitted is more toxic. It was found that the CO/CO_2_ weight ratio of the reference foam was significantly higher than foams modified by EPC. Its value was 0.32. The CO/CO_2_ weight ratio of EPC50 foam was reduced by 72% in comparison with the EPC0 foam. This was also due to the formation of a char layer on the surface of the polyurethane, which prevented further combustion and therefore a higher emission of toxic gases.

**Figure 5:**
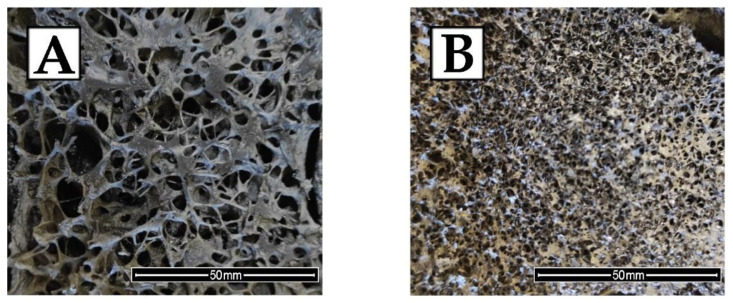
RPU/PIR foams after combustion in a cone calorimeter: (**A**) EPC0 foam; (**B**) EPC50 foam.

All obtained results suggest that the modification of RPU/PIR foams by EPC was able to effectively reduce the flammability of the obtained materials and the toxicity of gases emitted during combustion. Photographs of RPU/PIR foams without filler (a) and with the highest filler content (b) after combustion in the cone calorimeter are shown in Figure 5. 

Figure 5 shows a comparison of the surfaces of the foams without filler and with the highest filler content after the flammability test. Both surfaces were significantly different from each other. The unmodified foam (Figure 5a) did not form a char layer. Only the brittle skeleton of the polyurethane matrix remained after burning the EPC0 foam. On the other hand, it could be clearly seen after burning the EPC50 foam (Figure 5b) that a thick, compact, char layer had formed on the surface of the foam. The char layer of modified foam stopped the degradation of the polyurethane matrix and provided excellent fire protection. Polyurethane foam, like most porous materials, is susceptible to combustion and has a tendency to rapidly spread flame, and its LOI value can be as low as 19% [49]. The LOI value for the EPC0 foam was 19.1%. The use of bio-filler increased this parameter from 19.9% for the EPC10 foam to 22.5% for the EPC50 foam. Increasing the LOI value with the increase in the amount of EPC added to the foams proved the cooperation of phosphorus and nitrogen during the combustion process. It also confirmed the role of EPC as an effective flame retardant in polyurethane formulation. 

**Figure 6:**
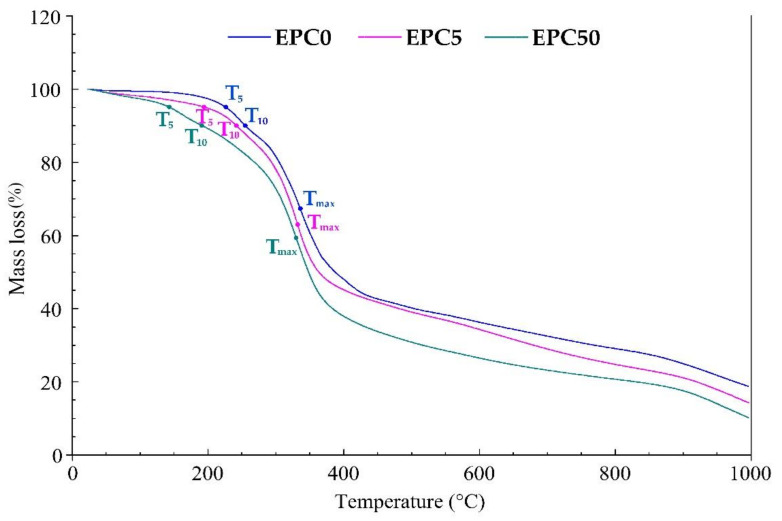
TGA curves of the EPC0, EPC5, and EPC50 foams.

Good thermal resistance is one of the basic requirements for insulation materials used in civil engineering. It is related to the physical and chemical changes in PU foam that occur under the influence of temperature on this material. The increased temperature degrades the polyurethane material because it breaks the weakest bonds in the PU matrix. The obtained RPU/PIR bio-composites were subjected to TGA testing in a nitrogen atmosphere in the temperature range of 20–1000 °C in order to characterize the effect of the addition of EPC on thermal resistance. The course of TGA curves and results of the TGA analysis of the reference foam (EPC0), foams with the lowest content of bio-filler (EPC5), and foams with the highest content of bio-filler (EPC50) are presented in Figure 6 and Table 6, respectively. Detailed TGA results for all obtained RPU/PIR foams are shown in Table S1 in the Supplementary Materials.

Figure 6 shows the characteristic temperatures: T_5_—temperature of 5% mass loss, T_10_—temperature of 10% mass loss, T_m_—temperature at which the highest mass loss occurred. The EPC0 foam showed higher thermal resistance than the modified foams. The mass loss did not start until about 160 °C. In the EPC0 foam, the highest mass loss occurred in the temperature range from 260 to 500 °C. The maximum rate of mass loss was 4.7%/min. The urethane bond dissociates in this temperature range. Decomposition of polyoxypropylene polyol fragments, carbodiimide and urea bonds, and the ether bond in polyol and isocyanurate rings also occurs in the same temperature range. Products in the solid, liquid and, to a large extent, gas phases were formed as a result of thermal degradation of the tested foam in an inert atmosphere. Ash (residue) in the amount of 21% of the initial mass was obtained after the end of heating. The mass loss of foams containing the minimum and maximum EPC content (EPC5 and EPC50) started at a temperature of about 50 °C. This was related to the decomposition of protein-based substances contained in the bio-filler. It proceeded rapidly up to approx. 350 °C (with a rate of up to 5.9%/min for EPC50). The loss of mass of EPC-modified foams was much slower after reaching this temperature. Then, its course was linear. Decomposition changes characteristic for RPU/PIR foams and EPC took place in the range of the highest mass loss. New solid, liquid, and gaseous products could be formed in the inert atmosphere as a result of the presence of decomposition products of both materials. It can be proved by the reduced amount of ashes (16% and 12% of the initial mass for EPC5 and EPC50, respectively) that remained after the end of the heating process.

**Figure 7:**
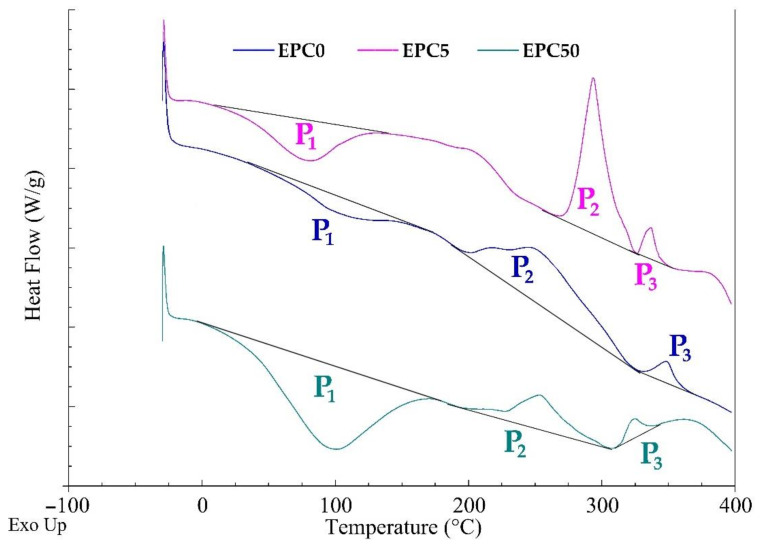
DSC curves of the EPC0, EPC5, and EPC50 foams.

Similar to the TGA analysis, Figure 7 shows the course of DSC curves of the EPC0, EPC5, and EPC50 samples. Detailed DSC results for tested RPU/PIR foams are shown in Table S2 in the Supplementary Materials. The differential scanning calorimetry test was carried out in the temperature range from −25 to 400 °C under a nitrogen atmosphere.

EPC0 foam is a polyurethane/polyisocyanurate reference foam used as a polymer matrix in this research. Three peaks were observed in the DSC curve of this foam, both endo- and exothermic. The first transformation (P_1_) was endothermic. It started at about 20 °C and lasted up to 110 °C. This was related to the diffusion of carbon dioxide (blowing agent) from the inside of the foam. The other two peaks indicated that the transformations from 260 °C to 320 °C (P_2_) and 325 °C to 340 °C (P_3_), respectively, were exothermic transformations. Many changes related to the decomposition of bonds present in the RPU/PIR foam occurred in these temperature ranges. These include the hydrolysis of the urethane bond, the degradation of the ether bond and polyoxypropylene fragments in polyol, the degradation of urea and carbodiimide bonds, or degradation of isocyanurate rings. However, these transformations are endothermic. Compounds containing hydroxyl and isocyanate groups could be obtained as a result of the hydrolysis of the urethane bond. Low-molecular substances containing OH groups could be also released during the decomposition of polyether polyol (Rokopol RF-551) chains. In turn, the degradation of isocyanurate rings leads to obtaining, for example, free isocyanates. The presence of compounds containing OH and NCO groups can lead to a rapid and highly exothermic chemical reaction. Thus, the measured energetic effect of the processes taking place is the sum of the endothermic effects of bond decomposition and the exothermic effects from the reaction of hydroxyl groups with isocyanate groups of the decomposition products. It was found based on the course of DSC curves that the exothermic effect was dominant [50,51]. 

The EPC5 and EPC50 foams contained the smallest (5 wt.%) and the highest (50 wt.%) content of EPC added into the polyurethane/polyisocyanurate foam. The presence of three transformation peaks was also noted, as in the case of the DSC curve of the reference foam without the bio-filler. The first (P_1_) and the third (P_3_) transformation took place in the same temperature ranges and did not show significant changes in the energetic effect in comparison with the unmodified foam (EPC0). This meant that analogous changes took place in the EPC-modified foams (e.g., diffusion of the blowing agent). The second transformation (P_2_) was more complex. Its total energetic effect depended on the reaction of the decomposition of individual bonds in the foam, the reactions of the OH groups with the obtained NCO groups, and the decomposition of organic substances contained in the EPC. It can be concluded based on the DSC curves that the exothermic effect was also dominant in modified foams. However, it was significantly lower than in the foam without a bio-filler.

## 3. Materials and Methods

### 3.1. Raw Materials

Rokopol RF-551 (product of sorbitol oxyalkylation, PCC Rokita, Brzeg Dolny, Poland) and Purocyn B (polymeric MDI, Purinova, Bydgoszcz, Poland) were used as polyol and isocyanate raw material to obtain rigid RPU/PIR foams, respectively. The polyether polyol and polyisocyanate met the requirements of ASTM D2849-69 and ASTM D1638-70.

The catalytic system used in the RPU/PIR foams synthesis consisted of anhydrous potassium acetate (Chempur, Piekary Śląskie, Poland) used in a 33% solution in diethylene glycol (Chempur, Piekary Śląskie, Poland) and DABCO (1,4-diazabicyclo[2,2,2]octane, Alfa Aesar, Ward Hill, MA, USA) was also used in a 33% solution in diethylene glycol (Chempur, Piekary Śląskie, Poland). Tegostab 8460 (polyether polydimethylsiloxane copolymer, Evonik, Essen, Germany) was used as the foam structure stabilizers. Antiblaze TMCP (chlorinated phosphate ester, Albemarle, Frankfurt, Germany) was used as a flame retardant. The RPU/PIR foams blowing agent was carbon dioxide generated in situ in the reaction of water with an excess of polyisocyanate raw material.

**Figure 8:**
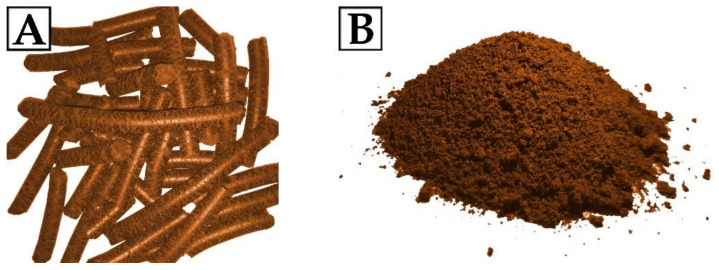
Evening primrose oil cake: (**A**) pellet, (**B**) ground pellet.

Evening primrose oil cake (EPC) from local oil mill (stuja.pl, Bydgoszcz, Poland) was used as a bio-filler. EPC in the form of pellets was mechanically ground in a lab grinder and dried (100 °C, 24 h) to remove any traces of water in the bio-filler. The ground oil cake was subjected to sieve analysis to determine the size of the filler particles. The size of the filler used for RPU/PIR foam synthesis was below 0.5 mm. The particle size is an important parameter that determines the even distribution of the particles in the polymeric matrix. It has been found that small particles can agglomerate to form large, dense clusters of filler in the polyurethane matrix. Both too small and too large physical filler particles result in the formation of materials with an inhomogeneous structure, which results in defects in the polyurethane matrix [52]. The moisture content, determined by the removal of water and measuring the weight loss was 1–1.5 wt.%. The physical form of the oil cake used in this research is shown in Figure 8. Chemical composition of the ground bio-filler (according to the information received from the manufacturer) is presented in Table 7.

Oil cake is a by-product of oil pressing. During cold pressing of evening primrose seeds, two fractions are obtained—oil and oil cakes (pomace). It is characterized by a high protein content, slightly lower content of fatty acids and crude fiber, and a high content of mineral salts. The level of fat content and the presence of crude fiber in the oil cake result from the efficiency of pressing the oil from evening primrose seeds while the content of protein, minerals, and vitamins depends on the genetic characteristics of this plant [53].

### 3.2. Preparation of Rigid Polyurethane/Polyisocyanurate Foams

Rigid polyurethane/polyisocyanurate foams were obtained at a laboratory scale using a two-component system (components A and B) and a one-step method. Preparation of the components and preparation of RPU/PIR foams were carried out at a temperature of 22 ± 2 °C. Several experimental studies were required to establish the optimal composition of additives (catalysts, surfactant, flame retardant, and blowing agent) to modify the formulation with a bio-filler. The basis for determining the amount of raw materials was the hydroxyl number of the used polyol. The amount of isocyanate raw material was selected taking into account the ratio of NCO groups to OH groups, which was 3:1 for RPU/PIR foams. The calculated amount of isocyanate raw material was increased by the excess of isocyanate necessary to carry out the reaction between distilled water and NCO groups to generate the blowing agent (carbon dioxide). In the next step, the amount of additive was determined by selecting their amount in the formulation in parts by weight per 100 parts by weight of polyol. The formulations of the synthesized RPU/PIR foams are shown in Table 8.

Formulations of 11 foams were developed. The obtained materials were marked as: EPC0—foam without bio-filler and EPC5–EPC50 foam with increasing percentage amount of bio-filler from 5 to 50 wt.% (increase in the amount every 5 wt.% in relation to the sum of polyol and polyisocyanate weights in each subsequent foam).

The foaming process of RPU/PIR foams is a very important stage in the technology of their production because it determines the properties of foams. The foaming process begins after mixing the polyisocyanate raw material (component A) with a polyol premix containing polyol raw material and all additives (component B). The combination of components A and B initiates a series of reactions where the end product is polyurethane. The indicator of the progress of the reaction is the change in color and consistency of the reaction mixture [54]. During the process of combining component A with component B, it is necessary to synchronize the moment of the maximum release of the blowing agent (carbon dioxide) with the time when the optimal viscosity is achieved. Too low and too high a viscosity of the reaction mixture makes the proper shaping of the cells impossible [55].

The reaction for obtaining polyurethane materials includes two basic processes taking place simultaneously. First is polyaddition of the reaction mixture and second is taking the shape of the finished product in the mold. A very important stage that affects the proper course of the polymerization is intensive mixing of the reaction mixture. The viscosity of polyol premixes was tested in order to better understand the effect of the bio-filler on the foaming process of RPU/PIR foams. The premix viscosity is a very important parameter during the foaming of polyurethane foams. It affects the processing parameters of the obtained foams and some of their properties. The results presented in Table 9 showed that the viscosity of the premixes modified by EPC increased after the addition of the bio-filler.

Increasing content of EPC in the foam formulation increased the viscosity of the premixes. It was found, based on the obtained test results, that the viscosity decreased with the increase in the shear rate. This meant that the prepared premixes were classified as non-Newtonian fluids. The obtained results are consistent with the results obtained by Kurańska et al. who observed an increase in the viscosity of microcellulose-modified premixes [56].

RPU/PIR foams were obtained according to the developed formulations (Table 8) using the one-step method in a two-component system. For this purpose, the polyol raw material was weighed with additives and the bio-filler (component B) in a polypropylene cup with a volume of 0.5 dm^3^ while the polyisocyanate raw material (component A) was weighed in the second cup. Component A was mixed with component B with a mechanical stirrer (1000 rpm) for 10 s. Then, the reaction mixture was poured into an open mold, where the process of free foam rise took place. An open mold with internal dimensions of 25 cm × 25 cm × 30 cm was used in these studies. The synthesis of each RPU/PIR foam was repeated twice. The obtained foams were thermostated for 4 h at 120 °C after removing from the mold. Then, foams were cut into standardized samples and subjected to further testing.

### 3.3. Test Methods

The viscosity of the polyol premixes was determined in accordance with ISO 2555 using a DVII viscometer (Brookfield, Dresden, Germany). Viscosity was measured in a function of shear rate (0.5–100 rpm) at ambient temperature.

The course of the foaming process was analyzed using an electronic stopwatch in accordance with ASTM D7487 13e^1^ [57].

RPU/PIR foams were subjected to a scanning electron microscopy (SEM) using a HITACHI S-4700 scanning electron microscope (Hitachi High-Technologies Co., Tokyo, Japan) with a NORAN Vantage microanalysis system. SEM micrographs of materials modified by EPC were taken in a parallel direction to the foam rise.

The apparent density of the tested foams was determined as the ratio of the foam mass to its geometric volume in accordance with ISO 845:2006.

Compressive strength tests were carried out on the universal testing machine 5544 (Instron, Norwood, MA, USA) in accordance with ISO 844:2016. The maximum force causing a 10% decrease in the height of the foam in relation to the initial height was determined. Measurement was carried out in the direction of foam rise.

Brittleness (B) of the obtained foams was determined in accordance with ASTM C-421-61. Brittleness was calculated as the percentage weight loss of 12 normalized foam cubes in relation to their initial weight. The measuring device used for testing was a cubic chest made of oak wood with dimensions of 190 mm × 197 mm × 197 mm, rotating around its axis at a rate of 60 rpm. The chest was filled with 24 oak cubes with dimensions of 20 mm × 20 mm × 20 mm. The brittleness of the RPU/PIR foams was calculated from Equation (1):

(1) $$B=\frac{m_{1}-m_{2}}{m_{1}}\;\cdot 100\%$$

where *m*_1_—weight of samples before testing (g); *m*_2_—weight of samples after testing (g).

The change in linear dimensions (Δ*l*) of the tested RPU/PIR foams was determined after 48 h of thermostating at 120 °C (393 K) in accordance with ISO 1923:1981. The foam samples were measured in the direction of the foam rise. The Δ*l* was calculated from Equation (2):

(2) $$\Delta l=\frac{l-l_{0}}{l_{0}}\;\cdot 100\%$$

where *l*_0_—sample length before thermostating (mm); *l*—sample length after thermostating (mm).

The mass loss (Δ*m*) of the tested foams was determined after 48 h of thermostating at 120 °C (393 K) in accordance with EN ISO 4590:2016-11. Δ*m* of RPU/PIR foams was calculated from Equation (3):

(3) $$\Delta m=\frac{m_{0\;}-m}{m_{0}}\;\cdot 100\%$$

where *m*_0_—sample mass before thermostating (g); *m*—sample mass after thermostating (g).

The change in geometrical volume (Δ*V*) of the tested foams was determined after 48 h of thermostating at 120 °C (393 K) in accordance with ISO 1923:1981. Δ*V* was calculated from Equation (4):

(4) $$\Delta V=\frac{V-V_{0}}{V_{0}}\;\cdot 100\%$$

where *V*_0_—geometrical volume of sample before thermostating (mm^3^); *V*—geometrical volume of sample after thermostating (mm^3^).

The thermal conductivity of the RPU/PIR foams was determined by testing the thermal conductivity coefficient (*λ*). The FOX 200 apparatus (TA Instruments, New Castle, DE, USA) was used to carry out these studies. It allowed us to determine the value of λ in the range of 20–100 mW/(m·K). Measurements were performed in series, every 0.5 s. The value of the thermal conductivity coefficient was determined from the Fourier Equation (5):

(5) $$q=-\lambda \;\cdot \;\frac{dT}{dx}$$

where *q*—density of the total heat flux (W/m^2^) transported along the path *x*; *λ*—thermal conductivity coefficient (W/m·K); and *dT/dx*—temperature gradient in the *x* direction (K/m).

The content of closed cells was determined in accordance with the PN-ISO 4590:016-11. Foam samples without structural defects were used for the tests. The measuring principle was based on the Boyle–Mariotte law. An increase in gas volume in a closed vessel caused a proportional reduction in pressure according to this law. If the volume of the chamber increases evenly in the presence of or without the sample, the pressure decrease will be lower in the case of an empty chamber. The method consists of determining the pressure change. Percentage amount of closed cell content in the sample (K_z_) was determined from Equation (6):

(6) $$K_{z}=\frac{V_{z}}{L\;\cdot \;w\;\cdot \;t}\cdot 10^{2}=\frac{V_{z}}{V_{c}}\cdot 10^{2}$$

where *V_z_*—closed volume of the sample read from the calibration graph (mm^3^); *L*—average length of the sample (mm); *w*—average width of the sample (mm); *t*—average thickness of the sample (mm); and *V_c_*—total volume of the sample (mm^3^).

Determination of absorbability and water absorption was carried out in accordance with DIN 53433. This method was based on measuring the weight of the sample before immersion and after 24 h of immersion in distilled water. Absorbability was calculated from Equation (7):

(7) $$A=\frac{m_{A}-m_{S}}{m_{S}}\;\cdot 100\%$$

where *A*—absorbability after 24 h immersion in distilled water (%); *m_A_*—sample weight after 24 h immersion in distilled water (g); and *m_S_*—weight of dry sample before immersion (g).

The method of determining the water absorption was based on measuring the weight of the sample after surface drying (determination of the water contained inside the foam after immersion). Water absorption was calculated from Equation (8):

(8) $$WA\;=\;\frac{m_{WA}-m_{S}}{m_{S}}\;\cdot 100\%$$

where *WA*—water absorption after surface drying (%); *m_WA_*—sample weight after surface drying (g); and *m_S_*—weight of dry sample before immersion (g).

Polyurethane foams modified by EPC were subjected to a controlled flux of thermal radiation (50 kW/m^2^) to analyze the rate of heat and smoke release by cone calorimeter (Fire Testing Technology, East Grinstead, UK). The flammability tests were carried out in accordance with ISO 5660:2015. Determination of fire parameters also consisted of measuring the oxygen concentration in the gases after combustion and the mass flow rate of these gases in the chimney. The following fire parameters were obtained as a result of the analysis:

— TTI—time to ignition, (s);
— THR—total heat release from the surface unit of the analyzed material, (MJ/m^2^);
— HLR—mass loss rate of sample/burning rate, (g/m^2^·s);
— HRR—heat release rate from the sample during combustion, (kW/m^2^);
— tHRR_max_—time to reach the maximum value of HRR (HRR_max_), (s);
— CO, CO_2_—emission of CO and CO_2_, respectively (kg/kg).

The limiting oxygen index (LOI) of the obtained foams was determined in accordance with ASTM D 2863-1970 standard. Concept Equipment (Poling, West Sussex, UK) apparatus was used for this measurements. It allowed us to determine the LOI value with an accuracy of 0.1%. The percentage limits of oxygen in a mixture consisting of oxygen and nitrogen sufficient to sustain the combustion of the sample were determined. LOI values were calculated from Equation (9):

(9) $$LOI=\frac{O_{2}}{O_{2}+N_{2}}\cdot 100\%$$

where *O*_2_—volumetric flow of oxygen at the limit concentration (m^3^/h); and *N*_2_—volumetric flow of nitrogen at the limit concentration (m^3^/h).

The thermal effects of the transformations taking place during the heating of the obtained RPU/PIR foams were tested by the DSC method under a nitrogen atmosphere in the temperature range from −40 to 400 °C using the differential scanning calorimeter DSC Q200 (TA Instruments, New Castle, DE, USA). Thermogravimetric analysis (TGA) of the RPU/PIR foams was performed under a nitrogen atmosphere in the temperature range from 20 to 1000 °C by an apparatus from TA Instruments (New Castle, DE, USA). In both analyses, the nitrogen flow was 2 cm^3^/min, the weight of the foam samples was in range from 8 to 10 mg, and the heating rate was 10 °C/min.

## 4. Conclusions

The results obtained in this study indicated that the addition of evening primrose (*Oenothera biennis*) oil cake to the formulation of rigid polyurethane/polyisocyanurate foams in any amount significantly influenced the morphology and functional properties of the modified foams. Modification of RPU/PIR foam by EPC improved most of the properties, which extended their application possibilities. The use of a bio-filler in the polyurethane system made it possible to obtain RPU/PIR foams with better performance properties than commercially available foams. The increase in the bio-filler content in polyurethane foams caused changes in the foaming process, slightly reducing the reactivity of the system. Modification of foams by EPC resulted in an increase in apparent density from 34.6 kg/m^3^ to 56.1 kg/m^3^ and the development of a more regular structure with a high content of closed cells (over 90%). The almost two-fold increase in apparent density resulted in an increase in the compressive strength of the obtained RPU/PIR foams (from 252.5 to 331.1 kPa) and their lower absorbability and water absorption. The addition of a bio-filler into the formulation made it possible to reduce the brittleness by about 10% and increased their resistance to temperature aging. The value of the thermal conductivity coefficient decreased from 34.6 mW/(m·K) for the reference foam to 27.4 mW/(m·K) for the foam with the highest EPC content. A very important observation from the application point of view was that the λ value decreased and the insulation properties of the modified RPU/PIR foams improved with the increase in the content of bio-filler in the foam formulation. The used bio-filler also influenced the flammability of the foams because it significantly increased their fire safety and reduced the amount of released heat and smoke.

It was found that the developed method allows for fast, cheap, and ecological management of evening primrose oil cake with the possibility of its reuse for another purpose—as a bio-filler improving the properties of RPU/PIR foams. Durable, low-brittle, flame-retardant, and eco-friendly bio-composite foams can represent a promising advance in the design of new functional materials that can be used in various industries. These studies are an example of the recycling of evening primrose oil cake, which is undoubtedly a big advantage from ecological and economic points of view.

## 5. Patents

The synthesis of RPU/PIR foams modified by oil cakes (pomaces) is protected by patent no. PL-227036.

## Figures and Tables

**Table 1:** Processing times of obtained RPU/PIR foams.

Sample	Cream Time (s)	String Gel Time (s)	Tack Free Time (s)	Free Rise Time (s)
EPC0	16 ± 1	32 ± 1	74 ± 1	66 ± 1
EPC5	16 ± 1	32 ± 1	75 ± 1	67 ± 1
EPC10	16 ± 1	32 ± 1	75 ± 1	68 ± 1
EPC15	16 ± 1	34 ± 1	76 ± 1	69 ± 1
EPC20	16 ± 1	34 ± 1	81 ± 1	75 ± 1
EPC25	16 ± 1	34 ± 1	84 ± 1	78 ± 1
EPC30	16 ± 1	35 ± 1	84 ± 1	78 ± 1
EPC35	16 ± 1	36 ± 1	89 ± 1	78 ± 1
EPC40	16 ± 1	36 ± 1	96 ± 1	80 ± 1
EPC45	16 ± 1	36 ± 1	98 ± 1	83 ± 1
EPC50	16 ± 1	38 ± 1	98 ± 1	83 ± 1

**Table 2:** Statistical analysis of SEM micrographs.

Sample	Cell Size (μm)	Wall Thickness (μm)	Content of Cells per Area Unit (Cell/mm^2^)
EPC0	319 ± 15	17 ± 2	10 ± 1
EPC10	260 ± 14	19 ± 2	11 ± 1
EPC20	250 ± 14	22 ± 2	11 ± 1
EPC30	240 ± 12	23 ± 2	12 ± 1
EPC40	230 ± 12	23 ± 2	13 ± 1
EPC50	204 ± 12	24 ± 2	13 ± 1

**Table 3:** Thermal insulation properties of RPU/PIR modified by EPC.

Sample	Thermal Conductivity Coefficient λ (mW/(m·K))	Closed Cells Content (%)	Absorbability (%)	Water Absorption (%)
EPC0	34.6 ± 0.1	80.9 ± 0.9	10.9 ± 0.2	5.5 ± 0.3
EPC5	34.2 ± 0.1	81.1 ± 0.9	10.9 ± 0.2	5.5 ± 0.3
EPC10	34.0 ± 0.1	83.0 ± 0.9	10.7 ± 0.3	5.1 ± 0.3
EPC15	33.6 ± 0.1	84.7 ± 1.0	10.7 ± 0.3	5.0 ± 0.3
EPC20	33.5 ± 0.1	88.6 ± 0.9	10.6 ± 0.5	4.8 ± 0.2
EPC25	30.6 ± 0.1	90.1 ± 0.9	10.6 ± 0.2	4.6 ± 0.2
EPC30	29.1 ± 0.1	90.6 ± 1.1	10.6 ± 0.6	4.2 ± 0.2
EPC35	28.8 ± 0.1	90.8 ± 0.9	9.0 ± 0.5	3.5 ± 0.2
EPC40	28.1 ± 0.1	90.8 ± 0.8	8.3 ± 0.5	2.2 ± 0.2
EPC45	27.7 ± 0.1	91.7 ± 1.3	8.0 ± 0.8	2.0 ± 0.2
EPC50	27.4 ± 0.2	93.1 ± 1.2	6.6 ± 0.2	1.8 ± 0.1

**Table 4:** Results of physico-mechanical tests and simulated aging tests.

Sample	Apparent Density (kg/m^3^)	Compressive Strength (kPa)	Brittleness (%)	Δ*m* (%)	Δ*l* (%)	Δ*V* (%)
EPC0	34.6 ± 0.4	252.5 ± 3.9	23.8 ± 0.9	4.0 ± 0.4	5.0 ± 0.4	3.0 ± 0.4
EPC5	38.9 ± 0.8	264.9 ± 1.4	23.1 ± 0.8	3.0 ± 0.4	4.3 ± 0.4	2.8 ± 0.4
EPC10	42.8 ± 0.7	273.5 ± 5.2	22.0 ± 0.7	2.6 ± 0.4	4.0 ± 0.4	2.4 ± 0.4
EPC15	44.5 ± 0.8	278.9 ± 6.6	21.4 ± 0.6	2.6 ± 0.4	3.6 ± 0.4	1.9 ± 0.4
EPC20	47.3 ± 0.8	284.3 ± 3.1	20.9 ± 0.6	2.1 ± 0.4	3.3 ± 0.4	1.5 ± 0.4
EPC25	49.0 ± 0.8	288.9 ± 3.9	19.7 ± 0.6	1.4 ± 0.4	3.1 ± 0.4	1.0 ± 0.4
EPC30	49.5 ± 0.8	295.9 ± 4.8	19.1 ± 0.5	1.4 ± 0.4	3.1 ± 0.4	1.0 ± 0.4
EPC35	50.8 ± 0.8	299.2 ± 1.6	17.0 ± 0.5	1.2 ± 0.4	3.1 ± 0.4	0.9 ± 0.4
EPC40	53.2 ± 0.8	304.7 ± 2.9	14.0 ± 0.4	1.0 ± 0.4	2.9 ± 0.4	0.7 ± 0.4
EPC45	55.6 ± 0.8	320.4 ± 9.3	14.8 ± 0.4	1.0 ± 0.4	2.9 ± 0.4	0.6 ± 0.4
EPC50	56.1 ± 1.1	331.1 ± 6.3	13.1 ± 0.4	1.0 ± 0.4	2.8 ± 0.4	0.6 ± 0.4

**Table 5:** Results of the flammability tests of selected RPU/PIR foams.

Sample	TTI (s)	THR (MJ/m^2^)	HLR (g/m^2^·s)	HRR (kW/m^2^)	tHRR_max_ (s)	Amount of Released CO (kg/kg)	Amount of Released CO_2_ (kg/kg)	CO/CO_2_ (-)	LOI (vol.% of O_2_)
EPC0	1.48	14.3	10.22	278.90	10	0.82	6.2	0.132	19.1
EPC10	1.53	7.7	7.03	241.21	17	0.18	1.93	0.093	19.9
EPC20	1.85	6.8	6.87	227.77	19	0.11	1.61	0.068	20.4
EPC30	1.96	6.0	6.02	201.99	23	0.09	1.57	0.057	20.8
EPC40	2.24	5.9	5.35	189.87	26	0.07	1.45	0.048	21.7
EPC50	2.61	5.6	4.87	175.29	29	0.05	1.38	0.036	22.5

**Table 6:** TGA results of the EPC0, EPC5, and EPC50 foams.

Sample	T_5_ (°C)	T_10_ (°C)	T_max_ (°C)	The Highest Mass Loss (%/°C)	The Highest Mass Loss Rate (%/min)	Residue (%)
EPC0	222	254	342	0.47	4.7	21
EPC5	196	243	337	0.47	4.7	16
EPC50	145	191	332	0.59	5.9	12

**Table 7:** Average chemical composition per 1 kg of EPC.

Components	Dry Weight (%)	Proteins (%)	Minerals (g)	Vitamins (mg)	Fat (%)	Crude Fiber (%)	Tanins (µM/g)
Amount	98.50	38.41 *	52.06 **	208.40 ***	6.40	9.20	14–17

* The total nitrogen content per 1 kg of evening primrose oil cake is 7.87%; ** Ca—23.6 g, P—13.9 g, Mg—4.6 g, K—9.4 g, Na—0.3 g, Fe—0.17 g, Zn—0.08 g, Cu—0.006 g; *** E—14.0 mg, B1—4.5 mg, B2—4.0 mg, B6—7.0 mg, folic acid—2.0 mg, PP—175 mg, biotin—1.9 mg.

**Table 8:** Formulation of RPU/PIR foams modified by evening primrose oil cake.

Sample	Rokopol RF-551 (g)	EPC (g)	Tegostab 8460 (g)	33% DABCO (g)	33% Potassium Acetate (g)	Antiblaze TCMP (g)	Distilled Water (g)	Purocyn B (g)
EPC0	66.80	0.00	5.40	3.17	7.96	53.90	3.15	250.60
EPC5	66.80	15.90	5.40	3.17	7.96	53.90	3.15	250.60
EPC10	66.80	31.80	5.40	3.17	7.96	53.90	3.15	250.60
EPC15	66.80	47.60	5.40	3.17	7.96	53.90	3.15	250.60
EPC20	66.80	63.50	5.40	3.17	7.96	53.90	3.15	250.60
EPC25	66.80	79.30	5.40	3.17	7.96	53.90	3.15	250.60
EPC30	66.80	95.20	5.40	3.17	7.96	53.90	3.15	250.60
EPC35	66.80	111.00	5.40	3.17	7.96	53.90	3.15	250.60
EPC40	66.80	126.90	5.40	3.17	7.96	53.90	3.15	250.60
EPC45	66.80	142.80	5.40	3.17	7.96	53.90	3.15	250.60
EPC50	66.80	158.70	5.40	3.17	7.96	53.90	3.15	250.60

**Table 9:** Viscosity of polyol premixes at different shear rates.

Parameter	Viscosity (mPa s)		
	0.5 rpm	10 rpm	50 rpm
EPC0	580	310	270
EPC5	620	330	290
EPC10	710	360	310
EPC15	790	380	345
EPC20	820	410	370
EPC25	880	440	390
EPC30	910	460	405
EPC35	940	490	420
EPC40	980	515	480
EPC45	1130	535	505
EPC50	1300	550	530

## Data Availability

Data are contained within the article.

## Supplementary Materials

The following are available online at https://www.mdpi.com/article/10.3390/ijms22168950/s1, Table S1: TGA results of foams modified by EPC and reference foam without EPC, Table S2: DSC results of EPC0, EPC5 and EPC50 foams.
