# Supplementary material for: Effect of Evening Primrose (Oenothera biennis) Oil Cake on the Properties of Polyurethane/Polyisocyanurate Bio-Composites
Source: Int J Mol Sci. 2021 Aug 19;22(16):8950. doi: 10.3390/ijms22168950 (PMC8396507; doi:10.3390/ijms22168950)
Supplement: Supplementary file 1 [file ijms-22-08950-s001.zip › ijms-1335647-supplementary.pdf]

# Effect of evening primrose (*Oenothera biennis*) oil cake on the properties of polyurethane/polyisocyanurate bio-composites

**Joanna Paciorek-Sadowska, Marcin Borowicz and Marek Isbrandt**

**Table S1.** TGA results of foams modified by EPC and reference foam without EPC.

| Sample | $T_5$ (°C) | $T_{10}$ (°C) | $T_{max}$ (°C) | The highest mass loss (%/°C) | The highest mass loss rate (%/min) | Residue (%) |
|---|---|---|---|---|---|---|
| **EPC0** | 222 | 254 | 342 | 0.47 | 4.7 | 21 |
| **EPC5** | 196 | 243 | 337 | 0.47 | 4.7 | 16 |
| **EPC10** | 188 | 232 | 335 | 0.49 | 4.9 | 16 |
| **EPC15** | 186 | 225 | 335 | 0.50 | 5.0 | 15 |
| **EPC20** | 181 | 218 | 335 | 0.52 | 5.2 | 15 |
| **EPC25** | 175 | 211 | 334 | 0.53 | 5.3 | 15 |
| **EPC30** | 168 | 207 | 334 | 0.54 | 5.4 | 14 |
| **EPC35** | 160 | 203 | 333 | 0.55 | 5.5 | 14 |
| **EPC40** | 153 | 199 | 332 | 0.57 | 5.7 | 13 |
| **EPC45** | 149 | 196 | 332 | 0.58 | 5.8 | 13 |
| **EPC50** | 145 | 191 | 332 | 0.59 | 5.9 | 12 |

**Table S2.** DSC results of EPC0, EPC5 and EPC50 foams.

| Foam | Peak P1 | | | | Peak P2 | | | | Peak P3 | | | |
|---|---|---|---|---|---|---|---|---|---|---|---|---|
| | $T_{onset1}$ (°C) | $T_{max1}$ (°C) | $T_{offset1}$ (°C) | $H_1$ (J/g) | $T_{onset2}$ (°C) | $T_{max2}$ (°C) | $T_{offset2}$ (°C) | $H_2$ (J/g) | $T_{onset3}$ (°C) | $T_{max3}$ (°C) | $T_{offset3}$ (°C) | $H_3$ (J/g) |
| **EPC0** | 41.2 | 98.5 | 155.1 | 68.2 | 191.1 | 249.2 | 322.1 | 55.9 | 322.9 | 347.4 | 361.5 | 9.9 |
| **EPC5** | 12.4 | 79.2 | 135.5 | 90.3 | 260.9 | 292.7 | 322.8 | 43.5 | 322.8 | 336.8 | 350.1 | 14.2 |
| **EPC50** | 2.9 | 99.9 | 176.1 | 29.5 | 185.2 | 254.1 | 305.7 | 20.1 | 306.5 | 324.8 | 340.9 | 4.9 |
